# Strain-dependent production of interleukin-17/interferon-γ and matrix remodeling–associated genes in experimental *Candida albicans* keratitis

**Published:** 2012-05-10

**Authors:** Yanli Zou, Hongbo Zhang, Hongxia Li, Hao Chen, Wengang Song, Yiqiang Wang

**Affiliations:** 1 Department of Immunology, Taishan Medical University, Taishan, China; 2 Shandong Provincial Key Laboratory of Ophthalmology, Shandong Eye Institute, Shandong Academy of Medical Sciences, Qingdao, China; 3 Department of Immunology, Qingdao University, Qingdao, China

## Abstract

**Purpose:**

The aim of this study was to investigate the role of genetic background in determining the development or prognosis of experimental fungal keratitis by comparing the disease courses and related molecules of experimental *Candida albicans* in two common mouse strains.

**Methods:**

After intrastromal inoculation of 1×10^5^ *C. albicans* blastospores into corneas of Balb/c and C57BL/6 mice, all mice developed typical keratitis. The disease was monitored using a slit lamp microscope and scored for comparison of symptoms. At desired time points, blood was collected and corneal homogenates were prepared for enzyme-linked immunosorbent assay measurement of interferon (IFN)γ or interleukin (IL)17. Other corneas were processed for histological evaluation, pathogen load measurement, or total RNA extraction, the last of which was subjected to reverse transcription in conjunction with real-time PCR to measure genes of interest in terms of collagens, matrix metalloproteinases (MMPs), and the tissue inhibitors of MMPs (TIMPs).

**Results:**

The infected corneas from the two strains presented different manifestations. Corneal transparency was less affected in Balb/c mice than in C57BL/6 mice, and Balb/c corneas contained fewer pathogens than C57BL/6 corneas during the measured period (10 days). In both strains, keratitis started to resolve around days 7–10, but C57BL/6 mice healed slower than Balb/c mice as indicated by disease presentation, histology, and pathogen burden assay. By day 7 post infection, pseudohyphae were rare but cellular infiltration remained intensive in both strains. The surface of the Balb/c corneas remained relatively intact and smooth, and C57BL/6 corneal lesions produced open erosion areas. Perforation was never seen in the current study setting. In both sera and corneas, IL17 expression increased earlier than IFNγ, and C57BL/6 mice produced higher IL17 levels and lower IFNγ levels than Balb/c mice. Compared with C57BL/6 mice, Balb/c corneas produced more MMP-2, Col3a1, and Col4a1, and less or equivalent TIMP-2 at all detected time points. They also produced more MMP-13, less MMP-8, MMP-9, and TIMP-1 at day 3 post infection, but less MMP-13, basically equivalent MMP-8, and more MMP-9 at later time points.

**Conclusions:**

The disease course of experimental *C. albicans* keratitis depends on the genetic background of the host animals. The balance between IL17 and IFNγ, as well as among the common injury- and wound healing–related proteins, may contribute to the pathogenesis of *C. albicans* keratitis. This study suggests that great variance of disease presentation should be expected for human subjects with *Candida* keratitis.

## Introduction

Infectious keratitis is among the leading causes of blindness in many parts of the world, with the reported causative pathogens varying depending on the demographic characteristics of the subjects studied and the specific examination standards used for pathogen typing, as well as other factors. In a study performed in New York in the 1980s, fungal keratitis accounted for about 1% of all observed infectious corneal diseases [1], while this number was 35% in Florida during that same period [2]. Although the risk factors of keratitis have been proposed in many epidemiology studies, the pathogenesis of fungal keratitis remains ill defined, especially at the level of molecular mechanisms, or when compared with the knowledge about the pathogenesis of bacterial or viral keratitis. Using an experimental *Candida albicans* keratitis (CaK) model in Balb/c mice, we previously reported that the adaptive response and immunological memory were induced in host animals [3,4]. Recently we showed that interleukin (IL)17 plays critical role in the initiation of experimental CaK (unpublished).

Since all immunological processes occur in the context of mismatching histocompatibility, and thus depend highly on genetic background, our previous findings concerning the adaptive immune responses in CaK implied that fungal keratitis development may differ in subjects with different genetic backgrounds. In fact, quite a few immune-mediated ocular disease models have demonstrated dependence on histocompatibility, such as in the cases of bacterial keratitis [5,6], blepharoconjunctivitis [7], and herpes simplex viral keratitis [8]. Similar strain dependence was also observed in ocular pathological processes that are not so closely related with immunity, such as oxygen-induced photoreceptor damage [9], hypoxia-induced retinal damage [10], growth factor–induced corneal lymphangiogenesis and angiogenesis [11,12], and dry eye [13]. In the field of fungal keratitis pathogenesis, both Balb/c [3,14,15] and C57BL/6 mice [16], and the knockout mice from these backgrounds, have been used in different studies, but a parallel comparison has been lacking. This makes it hard to predict the significance of host genetic background in the pathogenesis of fungal keratitis. In the current study, we compared the development and resolution of experimental CaK in these two mice strains, and tried to reveal the related mechanisms.

## Methods

### Experimental animals

Male, 8–10-week-old Balb/c (H-2^d^) and C57BL/6 (H-2^b^) mice were purchased from the Academy of Military Medical Sciences (Beijing, China). Before being recruited into experiments, all corneas were examined individually with a slit lamp microscope. All animal experiments were performed in accordance with the Guidelines on the Humane Treatment of Laboratory Animals (Chinese Ministry of Science and Technology, 2006) and the Statement for the Use of Animals in Ophthalmic and Vision Research. Only the left corneas were used for manipulation, with the right eyes used as untreated controls.

### *Candida albicans* keratitis models

The protocol used for the murine CaK model has been described elsewhere in detail [3]. In brief, *C. albicans*, strain MYA-2876 (ATCC, Manassas, VA) was cultured routinely following the Shandong Eye Institute Biosafety Code. Blastospores were harvested, washed, and suspended in a saline buffer at a concentration of 1×10^8^/ml. The corneas were pierced near the center with a 30 gauge needle to the depth of the stroma. A 33 gauge needle with a 30 degree bevel (Hamilton, Reno, NV) was used to inject 1 µl of blastospore suspension (1×10^5^ blastospores) into the center of the cornea. In the sham-infection group, the same volume of saline buffer was substituted for the fungal suspension.

To ensure the accuracy and consistency of performance required for parallel comparison in different mice, intrastromal injection in all experiments was performed by the same individual (H.Z.) with good practice. The corneas were monitored daily using a slit lamp equipped with a digital camera, and photographed on desired days. A 12-point scoring system based on area and intensity and extent of the corneal opacity [17] was adopted to evaluate the development of the disease. At the desired time points, blood was collected from individual mice via tail venipuncture and used for enzyme-linked immunosorbent assay (ELISA) measurement of cytokines. Some mice were euthanized, and the corneas were harvested using a 2 mm diameter trephine; these samples were subjected to histological study, pathogen burden assay, or mRNA extraction, as described below. If not stated otherwise, at least three animals were included for each assay at each time point. All experiments were conducted at least twice.

### Histology

The corneas were immediately fixed in 4% formaldehyde (pH 6.9), embedded in paraffin wax, and serially sectioned into 4 μm sections. Neighboring sections were subjected to hematoxylin and eosin (H&E) staining and periodic acid–Schiff (PAS) staining following routine histological processing. The severity of the disease could be semiqualitatively evaluated by estimation of the affected area, cellular infiltration, hyphae distribution, and regularity of the corneal structure. For immunohistochemical labeling, corneas were harvested into optimal cutting temperature compound (Sakura Finetek USA, Inc., Torrance, CA), cryosectioned, and fixed with acetone. Sixty minutes’ staining at 37 °C with 5 μg/ml rat antimouse IL17 (BioLegend, San Diego, CA) was followed by three washes with phosphate buffer saline (0.01 M, pH 7.4) with 0.05% Tween-20 (PBS-T). Then, overnight staining with 10 μg/ml of fluorescein isothiocyanate–conjugated goat antirat IgG (Santa Cruz Biotechnology Inc., Santa Cruz, CA) in combination with 10 μg/ml of PE-conjugated antimouse CD4 (BioLegend) was performed at 4 °C and followed by three washes with PBS-T. The sections were viewed using a fluorescence microscope (E800; Nikon, Tokyo, Japan).

### Pathogen burden assay

Each harvested cornea was homogenized in 0.5 ml buffer (0.1 M Tris-HCl pH 8.0, 0.02 M EDTA in distilled water) using a Tissue-Tearer (Biospec Products, Bartleville, OK) at 18,000 rpm for 30 s. The homogenates were aliquoted, and two 10-fold dilutions (1:10 and 1:100) were prepared in PBS. The dilutions were chosen based on pilot experiments. One hundred microliter aliquots of each dilute were spread on 90 mm Sabouraud’s dextrose agar plates in triplicate. The plates were incubated at 37 °C for 48 h, and the plates that gave clearly isolated fungal colonies were used to count the numbers of colonies, which were later converted to a pathogen load in the whole cornea.

### Enzyme-linked immunosorbent assay for cytokines

The levels of interferon (IFN)γ and IL17 in corneal homogenates and sera were assayed using Mouse ELISA MAX^TM^ Deluxe Sets for IFNγ or IL17A (BioLegend), respectively, according to the protocol supplied by the manufacturer. Standard curves were prepared at the same time and used for calculation of the cytokine concentrations in samples. The readings for corneal homogenates were then converted to total gross amount of cytokines in each cornea.

### Reverse transcription and real-time polymerase chain reaction

To measure the expression of interested genes at mRNA level, reverse transcription followed by real-time quantitative polymerase chain reaction (RT-qPCR) was performed using the Taqman method. We focused on several matrix metalloproteinases (MMPs), the tissue inhibitors of MMPs (TIMPs), and the collagens that have been reported to be involved in the pathogenesis of corneal injury and wound healing (Table 1). In brief, at the desired time points, corneas were excised using a 2 mm diameter trephine and placed in ice-cold TRIzol reagent (Invitrogen, Gaithersburg, MD) with two corneas from each model pooled into one sample. Six corneas were used to generate three samples at each time point. The untreated corneas from the same mice were used as naïve controls. Total RNA was extracted and purified routinely, and reverse transcribed into cDNA using a PrimeScript RT Reagent Kit (Takara, Shiga, Japan), all following the manufacturer’s instructions. PCR was run in triplicate for each sample. Primer and probe sets are listed in Table 1 with ribosomal protein L5 (*RPL5*) as reference gene. Cycling conditions were as follows: 10 s at 95 °C and 45 cycles of amplification for 15 s at 95 °C and 1 min at 60 °C. After analysis with the endorsed software, the fractional cycle number for threshold fluorescence (threshold cycle, Ct) for each reaction was obtained. The average of three duplicates was used to calculate the relative Ct against RPL5 (ΔCt=Ct_gene_-Ct_RPL5_) for each sample. Then, the average ΔCt for the three samples in the CaK and control groups was used to calculate the ΔΔCt of the CaK samples (ΔΔCt=ΔCt_CaK_-ΔCt_control_). The relative expression folds of the CaK samples over controls were calculated as 1/2^ΔΔCt^.

**Table 1:** Primers and probes used for RT-qPCR assay.

**Gene symbol (accession number)**	**Primer and probe sequence (5′-3′)**	**Amplicon**
*MMP-2*	F: CTGGGAGCATGGAGATGGATA	96 bp
(NM_008610)	R: AAGTGAGAATCTCCCCCAACAC	
	P: FAM-ACATGCCTTTGCCCCGGGCA-TAMRA	
*MMP-8*	F: CAATTCCGGTCTTCGAGGAA	85 bp
(NM_008611)	R: TCCCAGTCTCTGCTAAGCTGAA	
	P: FAM-CCACGATGGTTGCAGAGAAGCTTAAAGA-TAMRA	
MMP-9	F: GGGTCTAGGCCCAGAGGTAA	86 bp
(NM_013599)	R: AGACACGCCCCTTGCTGA	
	P: FAM-CCACGTCAGCGGGCTTCTCCC-TAMRA	
*MMP-13*	F: AAGTGTGACCCAGCCCTATC	156 bp
(NM_008607)	R: CACATGGTTGGGAAGTTCTG	
	P: FAM-CTTCTGGCGCCTGCACCCTC-TAMRA	
*TIMP-1*	F: GGACCTGGTCATAAGGGCTA	175 bp
(NM_011593)	R: GGCATATCCACAGAGGCTTT	
	P: FAM-TCTGCGGCATTTCCCACAGC-TAMRA	
*TIMP-2*	F: CTGTCCCATGATCCCTTGC	87 bp
(NM_011594)	R: TGGTGCCCATTGATGCTCT	
	P: FAM-CATCTCCTCCCCGGATGAGTGCC-TAMRA	
*Col3a1*	F: GGCAGTGATGGGCAACCT	86 bp
(NM_009930)	R: GGTCCAACTTCACCCTTAGCA	
	P: FAM-CCCCCTGGCCCTCCTGGAACT-TAMRA	
*Col4a1*	F: ATTAGCAGGTGTGCGGTTTG	77 bp
(NM_009931)	R: CACTGCGGAATCTGAATGGT	
	P: FAM-AGCACCGGCCATGGTGATGGC-TAMRA	
*RPL-5*	F: GGAAGCACATCATGGGTCAGA	70 bp
(NM_016980)	R: TACGCATCTTCATCTTCCTCCATT	
	P: FAM-TGTGGCAGACTACATGCGCTACC-TAMRA	

### Statistical analysis

When necessary, statistical significance was determined by the Student *t* test, and by applying a 95% confidence interval (p<0.05) to judge significance.

## Results

### Differential courses of *Candida* keratitis in two mouse strains

**Figure 1:**
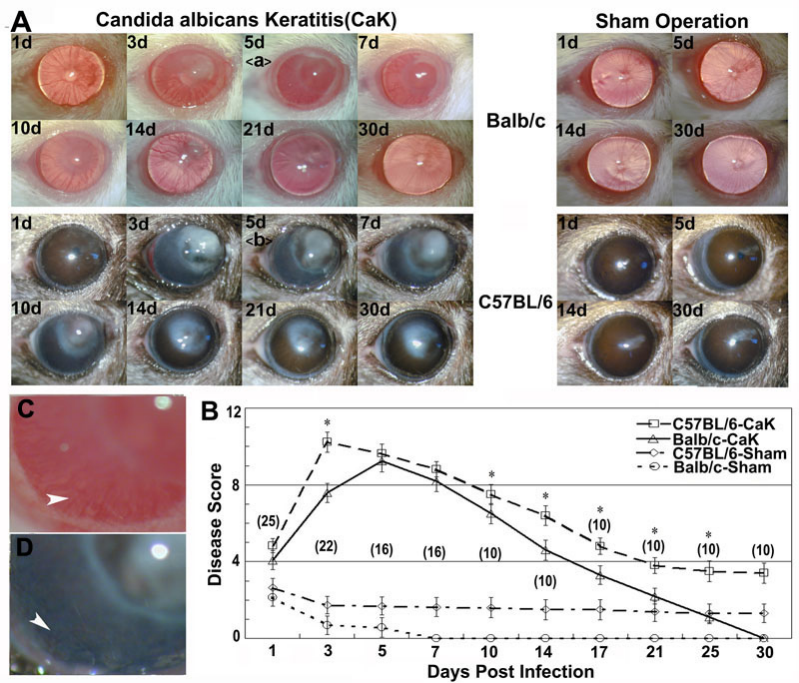
Differential courses of *Candida albicans* keratitis in Balb/c and C57BL/6 mice. Mice were inoculated with 1×10^5^ *Candida albicans* blastospores (CaK groups) or the same volume of saline buffer (sham infection). **A**: The corneas were monitored under slit lamp. The insets **C** and **D** showed details like transparency and neovascularization (arrow heads) at day 5. **B**: The diseases of the corneas were evaluated with the scoring system. The numbers of measured corneas in each group are given in parentheses in **B**.

With the optimized numbers (1×10^5^) of blastospore load, keratitis could be induced in all corneas in both strains, but the presentation of the disease differed in several ways (Figure 1). For example, during the severe period, the affected Balb/c corneas were characterized by forming a semitransparent bulb, with the areas beside the bulb also remaining semitransparent; in contrast, C57BL/6 corneas with CaK developed an irregular whitish mass and the areas beside the mass were also opaque. Heavy neovascularization was present in both strains, but this finding was not investigated in the present study. After the symptom apex around days 7–10, the diseases started to resolve in both strains, but Balb/c mice regained normal appearance of corneas earlier than C57BL/6 mice. Furthermore, while no abnormalities were seen in Balb/c corneas by day 21, an opaque area remained in C57BL/6 corneas. This area decreased slowly and lasted for at least one month beyond the observation period (data not shown). In sham controls, a similar but much smaller opacity was also present in most C57BL/6 corneas, but this was not found in any of the Balb/c mice (Figure 1).

### Histology and fungal burden in corneas with *Candida* keratitis

**Figure 2:**
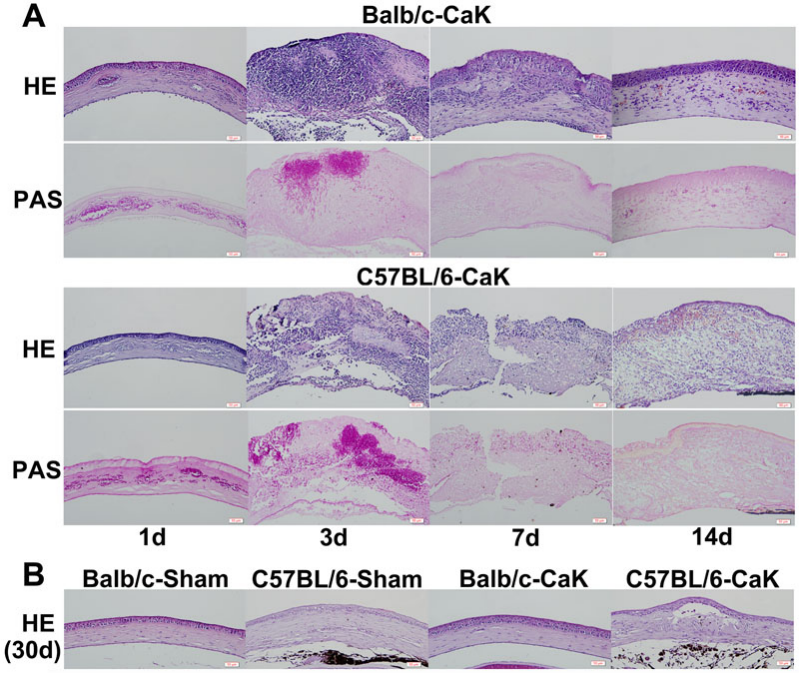
Histology of corneas on days 1, 3, 7, 14, and 30 post–*Candida albicans* keratitis induction. Five corneas belonging to each *Candida albicans* keratitis (CaK) group were serially sectioned at each time point and adjacent sections were stained using the hematoxylin and eosin (H&E) and periodic acid–Schiff (PAS) methods. H&E staining was mainly used for examination of the cellular distribution and gross structure of the cornea, and PAS staining for revealing the distribution of fungi. One representative section from each group at each time point is shown. **A**: Structures of infected corneas changed significantly with time. **B**: Lasting infiltration in corneas of both sham and infected C57BL/6 mice, but only transient infiltration in Balb/c corneas as indicated for day 30.

**Figure 3:**
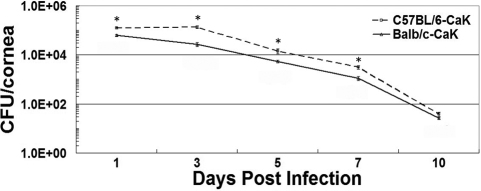
Quantification of *Candida albicans* in corneas using colony forming assay. Three corneas from each *Candida albicans* keratitis (CaK) group at each time point were included in the analysis. * p<0.05 (n=3) by Student *t*-test.

By histological examination, significant pseudohyphae and cellular infiltrates were found to be present as early as one day after inoculation in both mouse strains. By day 7 post infection, pseudohyphae were rare but cellular infiltration remained extensive in both strains. The surface of the Balb/c corneas remained relatively intact and smooth, while C57BL/6 corneal lesions showed an open erosion area (Figure 2). By day 14, unlike Balb/c corneas, cellular infiltration was still significant in the corneas of C57BL/6 mice. Starting from three weeks, the structure of Balb/c corneas was basically normal with occasional iris anterior adhesion, while C57BL/6 corneas contained multiple tunnels with cellular inner layers or even a separating diaphragm (Figure 2). No solid or granular residues were observed in any such blank spaces, implying that the content of these tunnels were fluid, and this might be the reason for the unresolved opacity in these corneas. Consistent with the PAS staining result, the colony forming assay also revealed higher pathogen burdens in the corneas of C57BL/6 mice than Balb/c mice (Figure 3). By 10 days, almost no detectable pathogens were present in the corneas.

### Interferon-γ and interleukin-17 in corneas and sera

**Figure 4:**
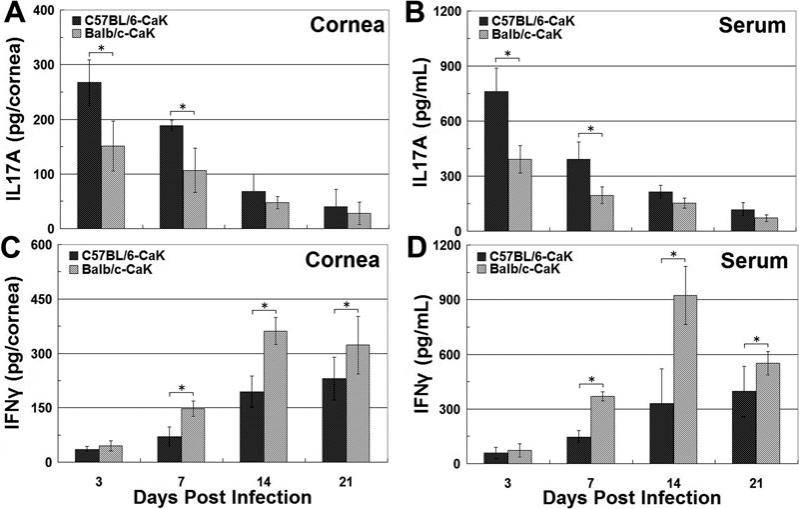
Interleukin-17A and interferon-γ production in corneas and serum during the first three weeks of *Candida albicans* keratitis induction. At different times post infection, the animals were sacrificed and sera were harvested. The corneas were removed using trephine and homogenized. The interleukin (IL)17A and interferon (IFN)γ in both serum and corneal homogenate were measured, and the amounts of cytokines in corneas were converted to the total amount in each cornea. **A**: IL17 contents in corneas. **B**: IL17 concentrations in serum. **C**: IFNγ contents in corneas. **D**: IFNγ concentrations in serum.* p<0.05 (n=3) for comparison in these two strains.

**Figure 5:**
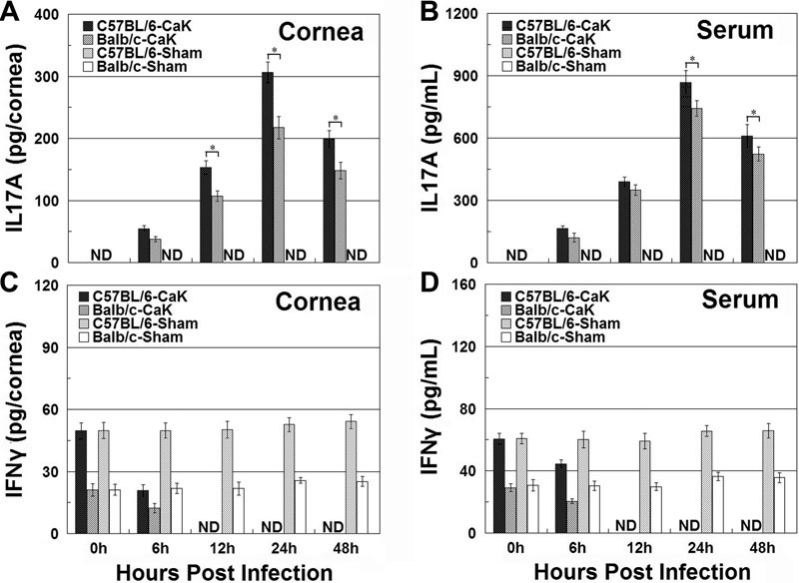
Interleukin-17A and interferon-γ production in corneas and serum during the first two days after infection or sham treatment. At 6, 12, 24, and 48 h post infection, the animals were sacrificed and the interleukin (IL)17A and interferon (IFN)γ in both serum and corneas were measured as described in the legend for Figure 4. Please note that no sham-infected corneas produced detectable IL17A at any time point in either strains. However, naïve mice of both strains had detectable baseline IFNγ production, and while sham treatment did not change the baseline level, *Candida albicans* keratitis (CaK) induction decreased IFNγ production to an undetectable level in the early phase. **A**: IL17 contents in corneas. **B**: IL17 concentrations in serum. **C**: IFNγ contents in corneas. **D**: IFNγ concentrations in serum. * p<0.05 (n=3) for comparison in these two strains.

**Figure 6:**
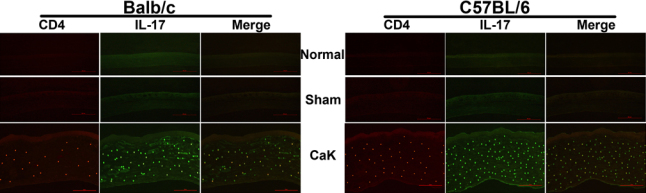
Production of interleukin-17 by CD4^+^ cells in corneas with *Candida albicans* keratitis. Mice were subjected to *Candida albicans* keratitis (CaK) induction and at day 1, the corneas were removed for double immunofluorescence. Cryosections were stained routinely for interleukin (IL)17 (green) and CD4 marker (red), as described in the Methods. Please note that not all IL17-producing cells are CD4^+^.

We previously showed that IL17 is a critical element in determining the initiation of experimental *Candida* keratitis, while IFNγ might be more important for disease resolution (unpublished). Figure 4 showed that upon induction of CaK, the levels of IL17 in both the sera and corneas of C57BL/6 mice were higher than in Balb/c mice at all time points. In contrast, IFNγ levels were always higher in Balb/c mice than in C57BL/6 mice (Figure 4). The early apex of IL17 levels and late apex of IFNγ in both mouse strains were also consistent with our earlier reports, and confirmed differential roles of these two cytokines in the pathogenesis of CaK. The production of these two cytokines in the early phase of CaK, specifically the first 48 h post infection, was also monitored with ELISA and immunohistochemistry. Consistent with the late onset of IFNγ motivation, the levels of IFNγ during the first two days remained at baseline level or even decreased to undetectable levels. In contrast, IL17 levels in naïve mice of both strains were undetectable and increased quickly upon CaK induction in both strains, reaching an apex at 24 h. Again, the levels of IL17 in C57BL/6 mice were significantly higher than in Balb/c mice in the early phase (Figure 5). Consistent with the differential levels of IL17 in the two strains, more IL17-producing cells, presumably CD4^+^ Th17 cells and neutrophils, were present in the corneas of C57BL/6 mice than in Balb/c mice, as indicated by the immunostaining performed 24 h after CaK induction (Figure 6).

### Differential responses of injury- and wound healing-related genes in the course of *Candida albicans* keratitis

**Figure 7:**
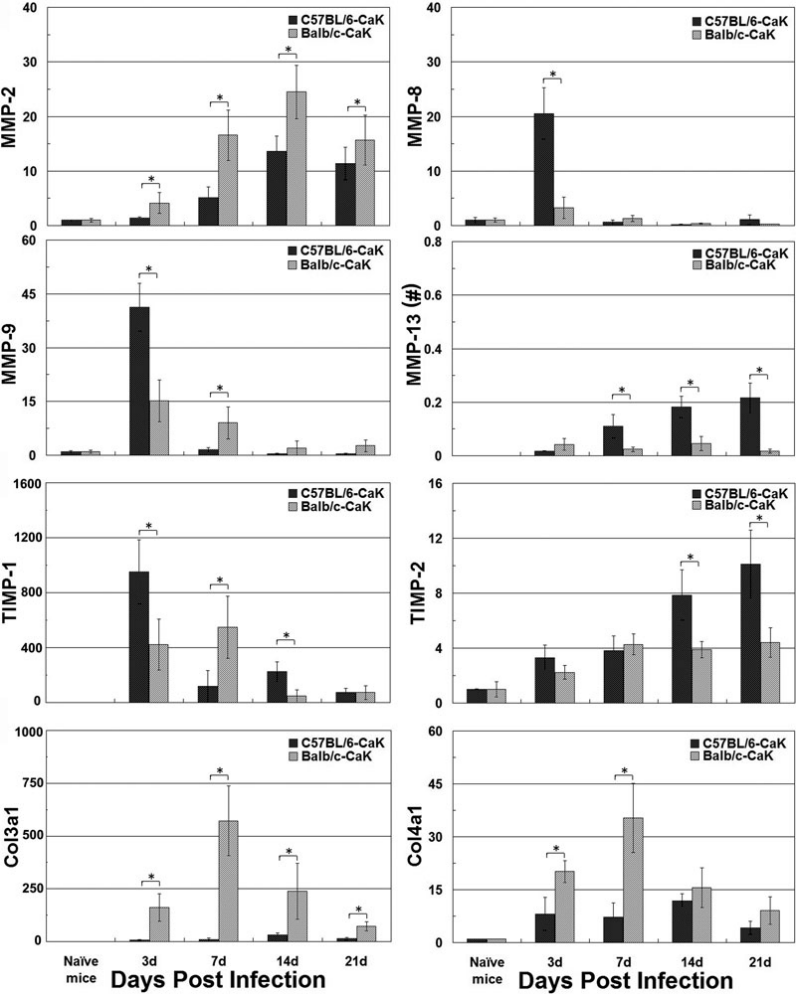
Gene expression in corneas as detected by reverse transcription and real-time quantitative polymerase chain reaction. The relative expression levels of each gene in the experimental groups over naïve mice were obtained by the ΔΔCt method, as detailed in the Methods section. Since the ΔCt for naïve mice was obtained by comparing Ct with that of reference gene ribosomal protein L5 (RPL5), the expression level for this group also has a standard deviation over 1.0. * p<0.05 (n=3) as compared between the two groups. Note (**#**): Since matrix metalloproteinase (MMP)-13 was not detectable in the naïve mice, with the result that the ΔΔCt method did not work, the relative levels of this gene compared with RPL5 in the experimental groups was obtained by the ΔCt method.

Lastly, we compared the changes of four MMPs, two TIMPs, and two collagen genes during the courses of CaK in the two mouse strains using RT-qPCR. It was found that all of the eight genes showed a changed pattern in a strain- and time-dependent manner (Figure 7). In brief, when compared with C57BL/6 mice, Balb/c corneas produced more MMP-2, Col3a1, and Col4a1, and less or equivalent TIMP-2 at all four detected time points. The changes in the other four genes were more complex, but also depended on the time points: At day 3 post infection, Balb/c mice produced more MMP-13 and less MMP-8, MMP-9, and TIMP-1, but this trend was reversed at three later time points.

## Discussion

Inbred mouse strains have been extensively used in immune-related studies, but great caution has to be taken when trying to extend experimental results obtained with one inbred mouse strain to another, let alone to the human species. For example, by profiling gene expression under *Pseudomonas aeruginosa* infections of the cornea in Balb/c and C57BL/6 mice, Huang et al. found that a gene may behave in concert or oppositely in different mouse strains [5], implicating that when two unrelated persons are challenged with the same pathogen, the expression of the same gene may increase in one person and decrease in another. In a previous study on chemical burn–induced corneal neovascularization, we documented that crystallins α, β, and γ were upregulated in Balb/c mice but downregulated in C57BL/6 mice at day 6 (unpublished). Our current data showed for the first time that two inbred mouse strains may manifest different presentations and prognoses when encountering the same amount of the same fungal pathogen via the same route. Considering the huge variations of conditions for human subjects in encountering fungal pathogens, we would expect various symptoms in actual patients bearing fungal keratitis. Correspondingly, the optimal treatments for these cases may also differ.

Comparing the CaK manifestations with histological or molecular findings in these two mouse strains suggested several factors involved in the pathogenesis of CaK. First, from the viewpoint of immune response, the sensitivities of mice to CaK may relate to differential levels of IL17 or IFNγ production, where IL17 dominates the pathogenic processes and IFNγ dominates the protection/recovery processes (Figure 4 and Figure 5). Many previous studies have classified immune responses as Th1- or Th2-dominant. With the recognition of Th17 immunoregulation [18,19], the understanding of involvement of Th17 in ocular diseases like uveitis [20,21] or infectious keratitis [22,23] is increasing. Beside conventional immune cells, the contribution of resident tissue cells to the modulation of IL17 must not be overlooked. It is known that specifically corneal epithelial cells modulate Th17 activity by producing a panel of cytokines [24-26]. Thus, in addition the strain-dependent function of immune compartment constituents like Th17, a difference in the immunomodulating efficacy of corneal cells in the two mouse strains may also contribute to the differential sensitivity of the mice to CaK.

The second issue concerning the differential courses of CaK is related with the broad concept of wound healing as reflected by the slower recovery of corneal structure in C57BL/6 mice than in Balb/c mice (Figure 1 and Figure 2). Histological study demonstrated that the long-lasting opacity in C57BL/6 corneas may be mainly caused by the presence of tunnels that were supposed to be filled with fluid in live corneas. This phenomenon might be caused by iris anterior adhesion, but other possibilities cannot be excluded, since some tunnels occurred in areas without iris adhesion, while other areas with iris adhesion had no tunnels (data not shown). In the sham-control C57BL/6 corneas, the opacity was caused by slight cellular infiltration (Figure 2). We propose that the differential wound-healing capability in CaK corneas of these two mouse strains was caused by different responses in the stromal layers but not in the epithelial layer, since the epithelium in both strains regained normal appearance by 30 days, when opacity was still obvious in C57BL/6 mice (Figure 2). In fact, another group even reported that the corneal epithelium of C57BL/6 mice heals faster than that of Balb/c mice [27]. Furthermore, our data showed a clear strain dependence of the expression of injury- and wound healing–related genes like MMPs, TIMPs, and collagens (Figure 7), although the implications of these changes remain unresolved in the current study. The early appearance of MMP-8, MMP-9, and TIMP-1 implied that these genes are more closely involved in injury response or early protection [28,29], and the gradual and continual increase of MMP-13 and TIMP-2 in C57BL/6 corneas suggest that these genes play roles in wound healing and matrix remodeling. However, the exact correlation between the overall molecular patterns and the clinical or histological manifestation was not investigated in this research, and more extensive studies are necessary to build up a paranormal picture of the MMP-TIMP interplay in the pathogenesis of fungal keratitis. Similarly, the matrix remodeling to rebuild the original structure of corneas also relates to the whole bunch of collagens, some of which are natural substrates of MMPs. Due to the changes in concert of Col3a1 and Col4a1 and the corneal manifestations in Balb/c mice, it is tempting to propose that these two collagens might play major roles in would healing in CaK corneas, as has been reported in other models [30,31]. However, more extensive studies on this topic using more rigorous methodologies such as gene knockout mice are warranted.

In summary, we observed differential disease courses in experimental CaK in two common mouse strains. At the molecular level, the immunological compartments concerning balance between IL17 and IFNγ are involved, as well as the matrix remodeling compartment concerning MMP-TIMP-collagens. Due to the huge biologic difference among various fungal pathogens, the impressions obtained here with *Candida albicans* are not necessarily applicable to other fungi. This being said, more research is necessary before a clear picture of the pathogenesis of fungal keratitis can be drawn. Again, when trying to translate laboratory observations concerning the pathogenesis of *Candida* keratitis obtained with inbred mice to the development of interfering protocols aimed at the prevention or treatment of *Candida* fungal keratitis in humans, the genetic dependence described in this study has to be borne in mind.
